# Recent Advances on Drug Development and Emerging Therapeutic Agents Through Targeting Cellular Homeostasis for Ageing and Cardiovascular Disease

**DOI:** 10.3389/fragi.2022.888190

**Published:** 2022-04-25

**Authors:** Tayyiba Azam, Hongyuan Zhang, Fangchao Zhou, Xin Wang

**Affiliations:** Michael Smith Building, Division of Cardiovascular Sciences, Faculty of Biology, Medicine and Health, The University of Manchester, Manchester, United Kingdom

**Keywords:** ageing, cardiovascular disease, autophagy, mitophagy, endoplasmic reticulum stress

## Abstract

Ageing is a progressive physiological process mediated by changes in biological pathways, resulting in a decline in tissue and cellular function. It is a driving factor in numerous age-related diseases including cardiovascular diseases (CVDs). Cardiomyopathies, hypertension, ischaemic heart disease, and heart failure are some of the age-related CVDs that are the leading causes of death worldwide. Although individual CVDs have distinct clinical and pathophysiological manifestations, a disturbance in cellular homeostasis underlies the majority of diseases which is further compounded with aging. Three key evolutionary conserved signalling pathways, namely, autophagy, mitophagy and the unfolded protein response (UPR) are involved in eliminating damaged and dysfunctional organelle, misfolded proteins, lipids and nucleic acids, together these molecular processes protect and preserve cellular homeostasis. However, amongst the numerous molecular changes during ageing, a decline in the signalling of these key molecular processes occurs. This decline also increases the susceptibility of damage following a stressful insult, promoting the development and pathogenesis of CVDs. In this review, we discuss the role of autophagy, mitophagy and UPR signalling with respect to ageing and cardiac disease. We also highlight potential therapeutic strategies aimed at restoring/rebalancing autophagy and UPR signalling to maintain cellular homeostasis, thus mitigating the pathological effects of ageing and CVDs. Finally, we highlight some limitations that are likely hindering scientific drug research in this field.

## 1 Introduction

Cardiovascular diseases (CVDs) are still the dominant cause of mortality and morbidity worldwide, with their prevalence increasing significantly over the years, especially in developed countries. The age-standardized global mortality of CVDs constantly increases yearly despite reduced morbidity due to increasing longevity. Ageing is considered a key risk factor for the development of CVDs. Heart failure prevalence increases nearly 15 times in the population over 80 years old compared with 20–39 years old adults (Benjamin et al., 2017).

Cardiac ageing features changes in cardiac structure and function, aggravated pathological hypertrophy and fibrosis, increased inflammatory responses and contributes to a functional decline in contractility. Numerous studies have identified that in the aged and diseased heart, a hallmarking feature is a disturbance in cellular homeostasis (Melser et al., 2013). There is an accumulation of damaged organelle as well as misfolded, unfolded, insoluble or damaged proteins both in the organelle and in the cytosol of cardiomyocytes which poses a proteotoxic threat to cell survival. This is particularly detrimental in the heart as cardiomyocytes are post-mitotic cells with a limited regenerative capacity. Disrupted cellular homeostasis also provokes the onset of CVDs. Therefore, to preserve cardiac function and increase survival, a number of well-orchestrated signalling processes aim to restore cellular balance by clearing the cell of unwanted proteins and organelle (Henning and Brundel, 2017). The evolutionarily conserved pathways involved in these processes include autophagy (Aman et al., 2021), mitophagy (Barbosa et al., 2019) and the unfolded protein response (UPR) (Koga et al., 2011).

These essential processes decline during aging, exacerbating disrupted cellular homeostasis. In recent decades, there have been significant advances in the understanding of the molecules and signalling pathways involved in these key events. As a result, novel targets for therapeutic intervention and drug development are beginning to emerge. Manipulating components among these signalling cascades can be achieved through pharmacological interventions. This holds the potential to restore cellular homeostasis within cardiomyocytes, promoting longevity and preventing pathological ageing and the onset of CVDs (Galluzzi et al., 2017).

## 2 Pathophysiological Processes Involved in Cardiac Ageing

All tissues in the body require oxygenated blood pumped by the heart. Hence, cardiovascular health is critical for the function of every single tissue as well as the longevity of the whole organism. However, it is inevitable that ageing is accompanied by a continuous decline in nearly most physiological processes, which makes the individual more susceptible to diseases and complications (Lakatta and Levy, 2003a). Therefore, ageing has a notable influence on the cardiovascular system, presenting as pathological remodelling, impaired left ventricular (LV) diastolic and systolic function, compromised endothelial function as well as increased arterial stiffness (Lakatta and Levy, 2003a; Lakatta and Levy, 2003b).

During ageing, there is a progressive decline in the number of cardiomyocytes in the heart leading to increased mechanical stress. To compensate, the remaining cardiomyocytes undergo hypertrophy resulting in an increased LV wall thickness. The hypertrophic cardiomyocytes present an increased nutrient and oxygen demand creating a slightly hypoxic environment resulting in increased reactive oxygen species (ROS) production (Meschiari et al., 2017). Cardiac ageing is associated with a decline in cardiac function demonstrated by a decreased fractional shortening, ejection fraction, heart rate and cardiac output (Antelmi et al., 2004), accumulating in a total decline in cardiac reserve capacity and life quality of the elderly (Strait and Lakatta, 2012). In addition, the early passive filling of the LV continues to decline while the left atrium tries to augment the late active filling. This results in enlargement and remodelling of the left atrium, which progresses to a decline in diastolic function of the aged heart (Lakatta and Levy, 2003a; North and Sinclair, 2012).

Furthermore, there is an increased deposition of extracellular matrix and fibrosis due to increased matrix metalloproteinases expression. Changes in the physical property of collagen, most likely due to non-enzymatic crosslinking, have also been reported in the aged heart. Cardiac fibroblasts are a key contributor to extracellular matrix production as they produce various extracellular matrix proteins such as collagen, α1-integrins, α2-integrins, α5-integrins and fibronectin (Meschiari et al., 2017). However, the underlying signalling mechanisms resulting in the change in cardiac fibroblasts to increase extracellular matrix production are unknown. Increased matrix metalloproteinases presence in the heart is associated with reduced angiogenesis ability. Ageing is accompanied by endothelial dysfunction due to impaired eNOS (endothelial nitric oxide synthase) activity and consequently reduced NO-dependent vasodilation (Collins and Tzima, 2011). Endothelial proliferation is also impaired in aged endothelial cells and is accompanied by reduced cellular migration and blurry cellular barriers (Swift et al., 1999; Weinsaft and Edelberg, 2001; Edelberg et al., 2002). This is partially attributed to reduced sensitivity to growth factors and facilitates extracellular matrix deposition, degradation of elastin fibres and accumulation of collagen fibres in vascular media (Sawabe, 2010). Moreover, increased peripheral vascular resistance in ageing individuals contributes to an increase in the stiffness of central elastic arteries, which subsequently reduces diastolic central pressures and imposes more afterload, further deteriorating cardiovascular health in the elderly (Nilsson et al., 2014).

Although the adult heart possesses limited regenerative ability, approximately 0.5–2% renewal rate per year, this percentage further declines with age. This implies an age-dependent decrease in the ability of the cardiac tissue to compensate for cardiomyocyte loss. This is also compounded with increased cell death in the heart (Li et al., 2020). Numerous animal studies have demonstrated that telomere shortening and damage are prominent features of cardiac ageing. Impaired telomeric DNA structure causes damage to the DNA, leading to cell cycle arrest and ultimately cell death (Li et al., 2020). Another key contributor to cardiac ageing includes an increased expression of pro-inflammatory factors such as IL-6, IL-1α and IL-1β. Another factor involved in cardiac ageing is an age-dependent alteration in Ca^2+^ signalling contributing to impaired cardiac relaxation and contraction. The sarcoplasmic reticulum Ca^2+^ pump (SERCA2) is involved in handling intracellular Ca^2+^ stores, however during ageing SERCA2 activity decreases (Janczewski and Lakatta, 2010).

Growing evidence from rodent models recapitulating features of human disease as well as aged mice and human samples have shown that there is an imbalance in cellular homeostasis within the myocardium. Genome-wide microarray as well as transcriptome and proteome analysis has revealed distinct differences in gene expression between young and old cardiomyocytes (Bernhard and Laufer, 2008). Numerous genes involved in autophagy, mitophagy and UPR signalling have shown an age-dependent decline in expression. This results in a reduced ability of the cell to maintain protein homeostasis (proteostasis) driving age-related cellular dysfunction. During ageing, cells become burdened with increasing loads of misfolded proteins and damaged organelle (Hipp et al., 2019).

## 3 Autophagy

Autophagy is an evolutionarily conserved pathway that plays a critical role in physiological and pathological cellular homeostasis. It is a form of intracellular degradation to destroy and recycle, damaged organelle and other cytoplasmic components. This cytoprotective mechanism ensures that there is appropriate organelle and protein turn-over to prevent excessive build-up of damaged organelles. Defective autophagy can lead to the endoplasmic reticulum (ER) stress and mitochondrial dysfunction leading to cell death (Aman et al., 2021). Overall, autophagy ensures the mitigation of cellular stress. There are three main types of autophagy, chaperone-mediated autophagy, micro-autophagy and macro-autophagy (Hansen et al., 2018).

During chaperone-mediated autophagy, selective cytosolic proteins expressing the amino acid motif KFERQ are recognised by chaperones such as the constitutive chaperone heat shock cognate protein of 70 kDa (HSC70). The protein is then transported into the lysosome *via* the lysosomal membrane protein LAMP2A for degradation (Cuervo and Wong, 2014). Approximately 30% of proteins are degraded in this manner (Alfaro, 2019). Micro-autophagy refers to the direct uptake of proteins required for degradation *via* invagination or protrusion of the lysosomal membrane (Schiattarella and Hill, 2016).

Compared to chaperone-mediated autophagy and micro-autophagy, macro-autophagy is the most prevalent form (Galluzzi et al., 2017). Macro-autophagy consists of a multi-step process: initiation, nucleation, cargo recognition and packaging, autophagosome expansion, autophagosome-lysosome fusion and cargo degradation (Yang and Klionsky, 2009). Autophagy initiation involves the assembly of the Unc-51-like kinase (ULK) complex consisting of ULK1, ATG101, ATG13 and focal adhesion kinase family interacting protein of 200 kDa (FIP200) (Hurley and Young, 2017). Under normal physiological conditions, the mammalian target of rapamycin complex 1 (mTORC1) phosphorylates ATG13 and ULK1 to prevent the induction of autophagy. Following stimulation, mTORC1 activity is inhibited leading to dephosphorylation of ULK1 and ATG13 (Yang and Klionsky, 2009). Concurrently, ULK1 undergoes autophosphorylation before phosphorylating ATG12 and FIP200. AMPK is also able to phosphorylate and activate ULK1. AMPK is capable of disrupting the interaction between ULK1 and mTORC1 but phosphorylating ULK1 at Ser^555^ (Wang et al., 2019a).

The ULK1-ATG13-FIP200-ATG101 complex phosphorylates various proteins including myosin-like Bcl-2 interacting protein 1 (Beclin-1), vacuolar protein sorting 34 (Vps34), Vps15 (also known as p150) (Cao et al., 2021). This is essential to aid in the transport of the class III phosphatidylinositol-3-kinases (PI3K) complex I to the isolated membrane. Subsequently, Vps34 phosphorylates phosphatidylinositol to induce the synthesis of phosphatidylinositol-3-phosphate (PI3P). Following the recruitment of the ATG2-WIPI (WD repeat protein interacting with phosphoinositides), a double membrane cup-shaped structure is formed called a phagophore at the phagophore assembly site. Studies have suggested that the phagophore arises from mitochondria and endoplasmic reticulum subdomians (Wang et al., 2019a).

The phagophore undergoes elongation to encircle all the cytosolic substances requiring degradation. There are two key ubiquitin-like conjugation systems involved in this step: the ATG12-ATG5-ATG16 complex and the microtubule associated protein 1 light chain 3 (LC3) system (Dikic and Elazar, 2018). ATG12 is first activated by E1 ubiquitin-activating enzyme ATG7 before being transported to E2 ubiquitin-conjugating enzyme ATG10 and conjugated to ATG5. The ATG12-ATG5 conjugate subsequently binds to ATG16 and undergoes self-oligomerisation into the ATG12-ATG5-ATG16 complex (Su et al., 2020). The LC3 system involves ATG4-mediated cleavage of the LC3 precursor protein LC3-I. E1 enzyme ATG7 and E2 enzyme ATG3 activate LC3-I before it conjugates to phosphatidylethanolamine (PE) to form the membrane associated LC3-PE (LC3-II) complex. Conjugation of ATG8 to PE at the C-terminus to yield ATG8-PE further promotes phagophore elongation. The sealed phagophore (now known as an autophagosome) undergoes maturation which involves stripping and clearing of the ATGs from the autophagosome outer membrane (Wang et al., 2019a).

**FIGURE 1:**
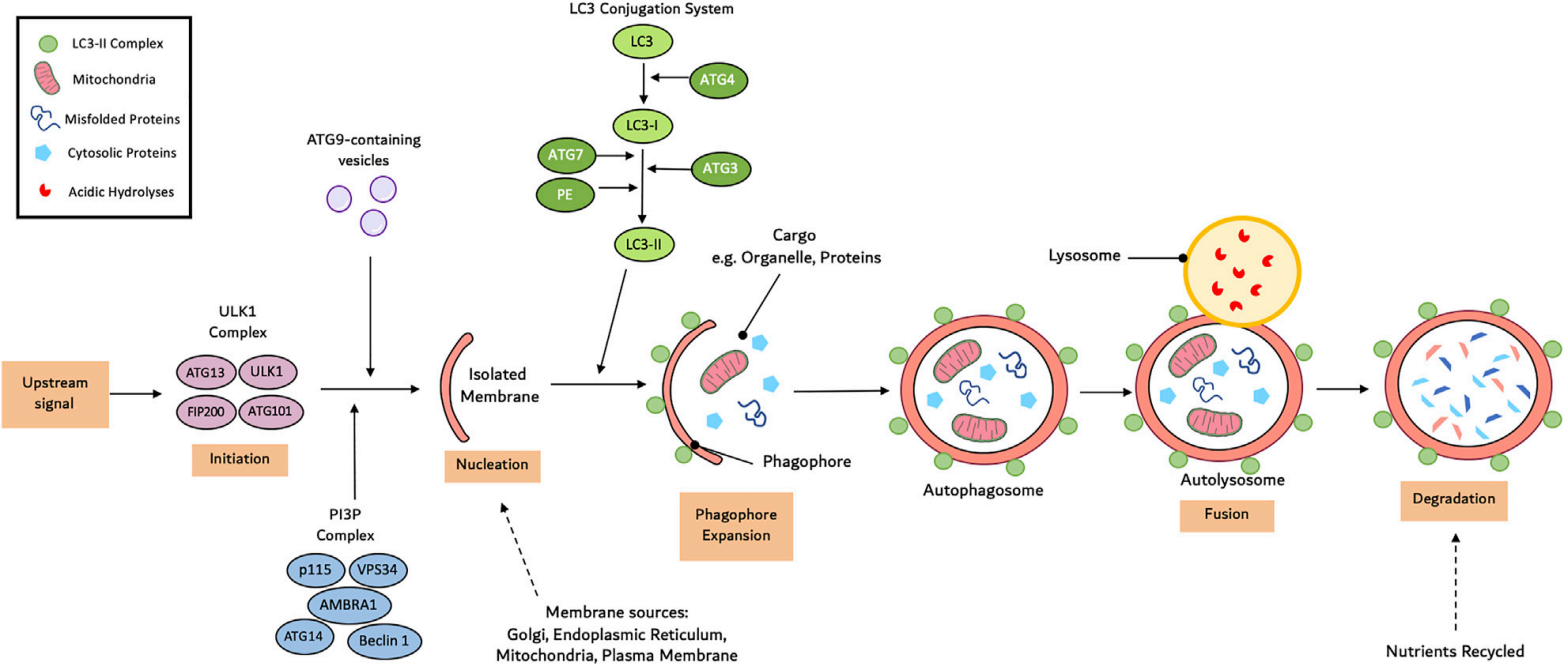
Mechanism of autophagy. Autophagy initiates with the formation of an isolated membrane/phagophore. Coordinated action of core autophagy machinery proteins results in the expansion of though phagophore into an autophagophore, which surrounds the cargo. Next, fusion with the lysosome result in the formation of the autophagolyososme. Lastly sequestered material is broken down inside the autophagolyosome and recycled.

During the final stage of autophagy, the autophagosome fuses with the vacuole/lysosome to release the inner single membrane vesicle containing the internal vesicles for degradation and recycling. Membrane tethering complexes, RAS-related GTP-binding protein (RAB) GTPases and soluble-N-ethylmaleimide-sensitive factor attachment protein receptors (SNARE) proteins have been linked to this process (Yim and Mizushima, 2020). These systems transport the autophagosome to the perinuclear region where they fuse with lysosomes and endosomes. Inside the autophagolysosome, lysosome-derived hydrolase such as cathepsin L and proteinase A and B degrade the cellular proteins and organelle into simple molecules such as amino acids that are then released back into the cytoplasm as substrates for biosynthesis (Hurley and Young, 2017). A summary of this processes can be seen in Figure 1.

### 3.1 Autophagy in Cardiac Ageing

Autophagy is critical in the maintenance of cardiac function; however, its activity decreases during cardiac ageing. Genetic modification of autophagy has unmasked its involvement in aging. For example, cardiac-specific deletion of ATG5 in mice caused no alternation in the survival rate, cardiac function and structure at 10-weeks old, however mice died at 24-weeks old and presented with a significant increase in age associated cardiomyopathy (Taneike et al., 2010). In contrast, another study showed overexpression of ATG5 in mice activated autophagy, extending the lifespan of the mice (Pyo et al., 2013). Similarly, cardiomyocyte-specific ATG7 knock-out mice displayed a heart failure phenotype, demonstrated by myofibrillar disarray, cardiac dysfunction and vacuolar cardiomyocytes (Li et al., 2016). Moreover, a recent study generated a mice model with Phe121Ala mutation of the Beclin 1, in which the interaction between Beclin 1 and its negative regulator BCL2 was impaired. This mice model showed elevated basal autophagic flux, reduced age-associated cardiac hypertrophy and fibrosis, as well as decreased cardiomyocyte apoptosis and delayed cardiac ageing (Fernández et al., 2018).

### 3.2 Molecular Pathways Underlying Reduced Autophagy in Cardiac Ageing

#### 3.2.1 Mammalian Target of Rapamycin

Mammalian target of rapamycin (mTOR) is a prominent nutrient and redox sensor in the body whose function is mainly embodied by mTORC1, which consists of mTOR, regulatory-associated protein of mTOR (Raptor), mammalian lethal with SEC13 protein 8 (MLST8), proline-rich Akt substrate of 40 kDa (PRAS40) and DEPTOR (Kim et al., 2002). This serine-threonine protein kinase participates in the regulation of various cellular processes, including protein synthesis and autophagy, primarily to maintain normal cardiac function. Moreover, mTOR inhibition in different organisms, through pharmacological interventions, exerts protection against a plethora of age-associated pathologies, thereby promoting longevity. The activity of mTOR is higher in both the aged mice (Hua et al., 2011) and human heart (Passtoors et al., 2013), leading to reduced autophagy activity, subsequently accelerating cardiac aging.

Consistently, inhibition of mTOR in mice by rapamycin both at early and the late stage is capable of extending lifespan (Harrison et al., 2009; Miller et al., 2011). In fact, reduced mTOR activity through rapamycin supplementation alleviated age-dependent cardiac hypertrophy and diastolic dysfunction in mice as well as reduced dysregulated mitochondria and ROS generation (Dai et al., 2014). The association between increased autophagy and reduced mTOR activity hints that the beneficial effect of rapamycin treatment could partially be explained by enhanced autophagic removal of damaged proteins and mitochondria. However, this requires further experimental verification since mTOR is involved in various cellular processes. The inhibition of the mTOR pathway through rapamycin is still a well-recognized modulation to protect against cardiac aging, in spite of possible side effects or controversial promotion of longevity.

#### 3.2.2 Insulin-Like Growth Factor 1/Akt Pathway

The activation of protein kinase B (PKB), also known as Akt, by growth factors including insulin-like growth factor 1 (IGF-1), promotes cell migration and proliferation. The IGF-1 associated pathway is initiated from the secretion of the growth hormone (GH) from the anterior pituitary gland, followed by that of IGF-1 from the liver. Various model organisms have shown the involvement of the GH/IGF-1 pathway in ageing and how the inhibition of this pathway promotes lifespan (Kenyon, 2011; Milman et al., 2016). The IGF-1 pathway is a critical regulator of longevity in mice and attenuated plasma IGF-1 levels correlated with extended lifespan in mice (Yuan et al., 2009). Meanwhile, another study carried on nonagenarians confirmed this correlation that the low IGF-1 level is a compelling predictor of life expectancy (Milman et al., 2014). Akt is a primary autophagy upstream inhibitor, and its activity increased with age. Many findings have built on the link between the Akt pathway in the heart and autophagy activity (Shiojima and Walsh, 2006; Miyamoto et al., 2008). Deletion of the IGF-1 receptor led to reduced Akt phosphorylation. Furthermore, mice presented with a reduced aging-induced hypertrophic response, decreased fibrosis and proinflammatory cytokines and increased autophagy, thus delaying the cardiac aging (Ock et al., 2016).

#### 3.2.3 AMPK-Activated Protein Kinase

The AMP-activated protein kinase (AMPK) is a master sensor of cellular energy supplements, that is, the ratio of AMP and ATP. Through the activation of a signalling pathway cascade, increased adenosine triphosphate (ATP) is produced and utilised more efficiently (Hardie and Carling, 1997). AMPK inhibits mTORC1 through the phosphorylation of tuberous sclerosis 1/2 (TSC1/2) and Raptor, resulting in increased autophagy activity (Gwinn et al., 2008). Moreover, AMPK directly induces autophagy through the phosphorylation of both ULK1 and Beclin-1 (Kim et al., 2011). During ageing there is a decline in AMPK activity in the heart (Slámová et al., 2016). In AMPK deficiency mice, disruption of mitochondrial ultrastructure and increased ROS production was observed. Meanwhile, treatment with an AMPK activator, Metformin, alleviated aging-associated cardiomyocyte contractile dysfunction (Turdi et al., 2010). However, the mechanisms by which metformin activates AMPK are still unclear and debatable. The treatment of metformin has been shown to extend health-span and lifespan through the activation of AMPK in mice with increased autophagy and attenuated aging-associated cardiac fibrosis, oxidative stress, inflammation and cardiac contractile dysfunction (Turdi et al., 2010; Martin-Montalvo et al., 2013).

#### 3.2.4 Sirtuins

Nicotinamide adenine dinucleotide dependent deacetylases (Sirtuins) family (comprising SIRT1-SIRT7) are another group of energy sensors conserved from bacteria to mammals, and their role in metabolism and ageing has been broadly studied. Sirtuins are localized in the nucleus, cytoplasm, and mitochondria and are extensively involved in the regulation of cellular metabolism (Houtkooper et al., 2012). SIRT1 has been reported to be involved in the regulation of autophagy and longevity in the heart. However, only low to moderate doses (up to 7.5-fold) of SIRT1 exerted beneficial effects seen as resistance to oxidative stress and apoptosis, while a high dose increased oxidative stress and age-associated cardiomyopathy (Alcendor et al., 2007). There are still many debates surrounding Sirtuins and their roles in cardiac aging. Irrespectively, the beneficial effects of Sirtuins in the promotion of longevity are widely admitted. *In vivo*, SIRT1interacted with essential components of the autophagy machinery, and thus increased the level of deacetylation for various Atg-related proteins like ATG5, ATG7 and ATG8 (Lee et al., 2008). Moreover, SIRT1 also negatively regulated the mTOR activity in a TSC2 dependent manner (Ghosh et al., 2010). Apart from SIRT1, some other members of the Sirtuins family have been studied in cardiac aging. As discussed above, IGF-1/Akt pathway is abnormally activated with age, and the sirtuin 6 (SIRT6), attenuated IGF-Akt activation through direct suppression of the promoter of IGF signalling-related genes (Sundaresan et al., 2012). SIRT3 has been reported to preserve mitochondrial function during ageing through the deacetylation of cyclophilin D (CypD) on lysine 166, and SIRT3-deficient mice showed accelerated signs of cardiac ageing such as cardiac hypertrophy and fibrosis at a young age and hypersensitivity to heart stress (Hafner et al., 2010).

### 3.3 Autophagy Targeting Drugs

The innate scavenger, namely autophagy declines during ageing while the pharmacological interventions that restore the autophagy activity in autophagy-deficient mice increase the longevity (Taneike et al., 2010). Hence, the importance of autophagy in cardiac ageing needs to be emphasised as well as its promising therapeutic role in clinical treatment. The induction of autophagy has shown beneficial effects in various diseases such as infection, autoimmune disease and cancer. Similarly, several autophagy inducers have shown cardioprotective effects in the heart (Galluzzi et al., 2017). A summary of the autophagy activators and their limitations can be seen in Table 1.

**TABLE 1:** Main activators of autophagy available to date and their limitations. AMPK, AMP-activated protein kinase, mTORC1, mechanistic target of rapamycin complex 1.

Drug name	Mode of action	Research progression	Major limitations	References
A-769662	AMPK activator	In preclinical development	indirect and nonselective	Cool et al. (2006)
BRD5631	Unknown	Unknown	Unknown mechanism	Kim et al. (2012)
Carbamazepine	Reduction in Ins (1,4,5) P3 and inositol levels	Approved for treatment of seizures and bipolar disorders	Inhibition in various neuronal functions	Lin et al. (2013)
Chloramphenicol	Unknown	Approved for second-line treatment of bacterial infections	Potentially mitochondriotoxic	Sala-Mercado et al. (2010)
			Unknown mechanism	
Everolimus	mTORC1 inhibitior	Approved for cancer therapy	Robust immunosuppressive effects	Dumortier et al. (2008)
			Inhibits many other autophagy independent mTORC1-dependent pathway	
Hydrogen sulfide	AMPK activator	Only allowed be used in experiments	Potentially toxic for the respiratory trait Unknown mechanism	Sun et al. (2015)
Lithium	Reduction in Ins (1,4,5) P3 and inositol levels	Approved for treatment of bipolar disorders	Inhibitions in multiple neuronal functions	Sarkar et al. (2008)
Metformin	AMPK activator	Approved for treatment of type II diabetes mellitus	Non-selective AMPK activator with various	Song et al. (2015)
			AMPK-unrelated effects	
Rapamycin	mTORC1 inhibitor	Approved for use in coronary stents and to treat a rare pulmonary disease	Has robust immunosuppressive effects and may cause mTORC2 inhibition when chronic administrated	Spilman et al. (2010)
Resveratrol	Caloric restriction mimetic	In clinical trials for treatment of several disorders	Potentially causes nephrotoxicity at high concentrations	Leontieva et al. (2013)
Spermidine	Caloric restriction mimetic	Nutritional supplement that is available over the counter	Producing ROS after degradation and potentially cytotoxic aldehydes	Eisenberg et al. (2009)
Trehalose	Unknown	In clinical trials for treatment of bipolar disorders, dry eye syndrome and vascular ageing	Unknown mechanism	Rodríguez-Navarro et al. (2010)
Trichostatin A	Unknown	Discontinued from clinical tests	Unclear mechanism, potentially linked to transcriptional effects	Shao et al. (2016)
Vorinostat	Unknown	Approved for cancer therapy	Unclear mechanism, potentially linked to transcriptional effects	Xie et al. (2014)

#### 3.3.1 Metformin

Metformin is a first-line drug prescribed to treat type II diabetes as it increases insulin sensitivity and decreases glucose output. However, recent studies have shown that Metformin also confers cardioprotective effects (Han et al., 2019). Metformin was found to significantly reduce morbidity in patients suffering from heart failure with preserved ejection fraction (Halabi et al., 2020). Furthermore, Metformin can improve the cardiovascular risk profile of patients suffering from ST-segment elevation myocardial infarction (Lexis et al., 2015). Recently, a number of studies have aimed to decipher the underlying signalling mechanisms by which Metformin confers cardioprotection and induces autophagy. Kanamori et al., 2019 used a model of δ-Sarcoglycan deficiency-induced dilated cardiomyopathy and treated the mice with Metformin for 4 weeks. Compared to saline treated mice, the authors found that Metformin reduced the extent of cardiomyocytes hypertrophy, interstitial fibrosis and cardiac dysfunction. Mechanistically, the Metformin-treated hearts displayed increased autophagy evident by increased autophagy flux and AMPK activation. Electron microscopy further validated these findings by revealing an increased number of autophagic vacuoles and lysosomes within the Metformin treated mice myocardium (Kanamori et al., 2019). Similarly, Metformin enhanced cardiac regeneration by increasing autophagy in cryoinjured zebrafish. Metformin increased cardiomyocyte proliferation and the vascular and endocardium endothelium (Xie et al., 2021a). Together these results suggest Metformin has clinical potential to enhance autophagy in the myocardium thereby preventing and attenuating cardiac stress. The cardioprotective effect of Metformin has not only been attributed to increased autophagy but various *in vivo* and *in vitro* studies have shown that its benefits correlated with reduced ER stress (Chen et al., 2019). Metformin treatment following Thapsigargin-induced ER stress markedly reduced the number of apoptotic cells in the mouse heart (Chen et al., 2017). A further study attributed the increased cell survival to the ability of Metformin to activate the PERK-ATF4 pathway to prevent cardiomyocyte death (Quentin et al., 2012). Furthermore, Cheang et al., revealed that Metformin improved endothelial function by increasing AMPK/PPAR signalling and reducing the ER stress response (Cheang et al., 2014).

#### 3.3.2 Rapamycin

Rapamycin is an FDA-approved immunosuppressant drug that inhibits the activity of mTOR—a key autophagy inhibitor. Various studies have shown that treatment with Rapamycin can extend lifespan by increasing autophagy (Abdellatif et al., 2018). To further investigate the role of Rapamycin in autophagy, Goa et al., generated a myocardial infarction-induced model of chronic heart failure. The rats were then either treated with Rapamycin or vehicle control. It was found that Rapamycin treatment reduced the extent of cardiac dysfunction and hypertrophy. Rapamycin also reduced the number of apoptotic cells both *in vivo* and *in vitro via* inhibition of mTOR resulting in increased autophagy (Gao et al., 2020). In another study of pressure-overload stress induced cardiac hypertrophy, it was demonstrated that Rapamycin decreased the expression of the Akt/mTOR/P70S6K1 pathway in a MEK/ERK/Beclin-1 signalling dependent manner (Gu et al., 2016). One of the major limitations of Rapamycin is that it is clinically used as an immunosuppressant, primarily to prevent organ rejection following transplantation. Nevertheless, it may be a valuable therapeutic tool in the prevention and treatment of CVD, but to validate this further research is required (Alnsasra et al., 2021).

#### 3.3.3 Spermidine

Spermidine is a naturally occurring activator of autophagy. It is a type of polyamine abundantly found in various foods such as soybeans, mushrooms, broccoli and rice bran (Tong and Hill, 2017). Previous studies have demonstrated that Spermidine is involved in cellular homeostasis and the stress response by regulating cellular growth, DNA stability, promoting protein deacetylation and cell survival. It has been shown to be a powerful activator of autophagy and it exhibits protective effects against the development of age-related diseases. It mainly exerts its action through inhibition of the transcriptional co-activator p300 which is a histone acetyltransferase. It is also capable of activating AMPK and inhibiting mTORC1 but to a much lesser extent (Frati et al., 2018). Recent studies have shown the cardioprotective role of Spermidine during CVDs. In a hypertrophic mouse model, spermidine decreased systemic blood pressure and the extent of cardiac dysfunction. It also prevented excessive hypertension-induced cardiac hypertrophy, ultimately delaying the progression of heart failure. Using cardiomyocyte-specific *Atg5* knockout mice, it was shown that the advantageous effects of Spermidine on increasing cardiomyocyte mitophagy and autophagy were repressed (Eisenberg et al., 2016). Spermidine also induces increased autophagic flux through the AMPK/mTORC1 pathway to exert beneficial effects following myocardial infarction (Yan et al., 2019).

#### 3.3.4 Trehalose

Trehalose is a natural non-reducing sugar, consisting of two α-linked glucose molecules. It is primarily synthesised by a number of organisms such as bacteria, yeast, insects and plants. Due to the lack of biosynthesis genes, mammals cannot synthesise Trehalose (Frati et al., 2018). Accumulating evidence, particularly over recent years indicates that Trehalose protects these organisms from a range of pathological stress, including oxidative stress, thermal stress and misfolded protein aggregation (Hosseinpour-Moghaddam et al., 2018). Following myocardial infarction, Trehalose induced autophagic flux, demonstrated by increased LC3-II, leading to improved cardiac function, and reduced cardiac remodelling, fibrosis, and apoptosis. These beneficial effects were significantly blunted in Beclin-1 heterozygous knock-out mice, which are unresponsive to autophagy inducers. Thus confirming that Trehalose plays a cardioprotective role by activating autophagy during an ischaemic insult (Sciarretta et al., 2018).

#### 3.3.5 Nobiletin

Nobiletin is a flavonoid that has gained scientific attention in the past few years. In streptozotocin (STZ)-induced diabetic cardiomyopathy in mice, Nobiletin blunted oxidative stress preserves cardiac function reflected by echocardiographic and haemodynamic measurements (Zhang et al., 2016). Furthermore, Nobiletin attenuates cardiac function and improves the survival rate following acute myocardial infarction in rats. This was parallel with alleviation in cardiac fibrosis, myocardial remodelling and cardiomyocyte apoptosis (Liu et al., 2021). Mechanistically, Nobiletin cardioprotective ability is *via* restoration of the autophagic flux by enhancing autophagosome conversion to autophagolysosome. However, it is suggested that the classical autophagy pathway may not be activated by Nobiletin as AKT, AMPK, Beclin-1 or mTOR expression was unchanged (Wu et al., 2017). Further research is required to decipher the mechanism by which the Nobiletin-mediated increase in autophagy is achieved. Nobiletin has also been linked to reduced oxidative stress demonstrated by increased cardiac SOD1 expression decreased NOX2 and NOX4 levels following pressure-overload stress (Zhang et al., 2017). The cardioprotective ability of Nobiletin has also been linked to reduced ER stress. Pressure-overload stress in the heart resulted in increased CHOP, cleaved/total caspase-12 and GRP78 in cardiac tissue. Nobiletin reversed these changes resulting in decreased cardiomyocyte cell death as evident by a reduction in the number of TUNEL-positive cells (Zhang et al., 2019). Together these studies suggest that Nobiletin can maintain cellular homeostasis by increasing signalling involved in autophagy, ER stress and oxidative stress.

#### 3.3.6 Sodium-Glucose Co-Transporters-2 Inhibitors

Sodium-glucose co-transporters-2 (SGLT-2) inhibitors are a group of drugs that were originally intended to treat diabetes mellitus (Zinman et al., 2015). However, SGLT-2 has recently become the topic of interest due to the benefits seen in heart failure patients. There have been five key trials investigating SGLT-2 inhibitors in diabetic patients. In the EMPA-REG (Empagliflozin Cardiovascular Outcome Event Trial in Type II Diabetes Mellitus Patients) Empagliflozin which was approved by the FDA in 2014 for the treatment of diabetes mellitus, showed that diabetic patients treated with Empagliflozin had significantly reduced cardiovascular-related mortality, reduced rate of heart failure related hospitalisation and reduced cardiovascular adverse effects (Zinman et al., 2015). Since this trial, a number of other SGLT-2 inhibitors have been shown to have cardioprotective properties, including, and Canagliflozin (Neal et al., 2017) and Dapagliflozin (McMurray et al., 2019). Originally, the studies were designed to assess the cardiovascular safety of the drugs, however, researchers discovered the unexpected benefit with respect to heart failure. Several studies employing mouse models have demonstrated the cardioprotective effects of SGLT-2 treatment. It can significantly improve cardiac function by reducing insulin resistance leading to improved sensitivity and increased glucose uptake, reduced systolic blood pressure without the presence of tachycardia, aiding in lipid ulitisation (Zhou et al., 2018) and attenuating cardiac cytokine expression (Trang et al., 2021). Scientists are now turning their attention to the underlying mechanisms by which SGLT-2 inhibitors are cardioprotective as they are yet to be completely established.

Numerous studies have linked SGLT-2 inhibitors with autophagy. SGLT-2 inhibitors are known to induce a state of starvation due to oxygen and nutrient deprivation in the body, triggering autophagy in various tissues including cardiomyocytes. However, the pathways by which SGLT-2 inhibitors stimulate autophagy are still unclear (Packer, 2020). Using a pressure-overload HF mouse model, Li et al., 2021 demonstrated that Empagliflozin treatment enhanced AMPK phosphorylation and subsequent activation and reduced mTORC1 expression. Together, this attenuated cardiac hypertrophy subsequent progression to heart failure (Li, 2021). Sayour et al., characterised the effect of canagliflozin in ischaemia-reperfusion injury. Non-diabetic rats were subjected to 30 min of left anterior descending artery (LAD) occlusion followed by 120 min reperfusion, before being injected with Canagliflozin. The study found that Canagliflozin treatment significantly decreased the level of serum troponin-T levels in the rats as well as reduced the myocardial infarct size. Cardiac function assessment revealed alleviated left ventricular diastolic and systolic dysfunction following myocardial ischaemia-reperfusion injury with Canagliflozin treatment compared to the vehicle control. This study also observed increased Akt and AMPK phosphorylation following treatment with Canagliflozin. NO production was also maintained due to the ability of Canagliflozin to enhance eNOS phosphorylation. The expression of numerous apoptotic markers was also reduced. *In vitro* experiments revealed increased endothelium-dependent vasodilation (Sayour et al., 2019). Together these studies suggest a link between SGLT-2 inhibitors and autophagy, however, more research regarding the mechanisms of autophagy induction is required.

#### 3.3.7 Irisin

Irisin is a novel proliferator-activated receptor-γ coactivator 1α-dependent myokine that originally was just considered an exercise-induced peptide that is released from skeletal muscle and the myocardium as a cleaved product of the type I membrane protein fibronectin type III domain containing 5 (FNDC5). Several recent studies have identified a therapeutic potential for Irisin in CVDs.

Irisin can alleviate pressure-overload induced cardiac hypertrophy by regulating autophagy. Following four-weeks of transverse aortic constriction (TAC), there was impaired autophagic flux and an accumulation of autophagosomes. This was reversed by Irisin supplementation which activated AMPK and ULK1 Ser^555^ (Li et al., 2018). Similarly, in a model of angiotensin II-induced cardiac hypertrophy, Irisin supplementation increased LC3II expression while P62 expression decreased. Mice also displayed alleviated myocardial hypertrophy and reduce cardiomyocyte cell death following pressure-overload induced stress (Li et al., 2019a). In addition, Lu et al., found that Irisin improved cardiac function following ischaemia/reperfusion injury, demonstrated by reduced cardiomyocyte apoptosis, infarct size and reactive oxygen species production. Ischaemia/reperfusion injury caused an upregulation in the expression of XBP1, GRP78 and CHOP, however this was reversed by Irisin treatment (Lu et al., 2020). This indicates that Irisin can regulate various signalling processes to maintain cellular homeostasis.

#### 3.3.8 Ivabradine

Ivabradine is the only approved hyperpolarisation-activated cyclic nucleotide-gated channel 4 (HCN4) inhibitor that is used clinically to treat chronic heart failure and stable angina. This drug specifically and selectively disrupts the cardiac pacemaker ion current flow, thus prolonging diastolic depolarisation and reducing the heart rate. This results in decreased myocardial oxygen demand thereby increasing coronary artery perfusion without hindering the cardiac muscle contraction force (Maagaard et al., 2019). Ivabradine specifically targets a sodium-potassium inward current channel in the sinoatrial node known as the HCN4 channel. Although it was approved in 2015, scientists are now uncovering other therapeutic benefits of this drug (Tse and Mazzola, 2015).

A number of studies have demonstrated that Ivabradine exerts a cardioprotective effect during myocardial infarction and cardiac hypertrophy. Short-term Ivabradine treatment increased exercise capacity and improved the left ventricular filling pressure following exercise in heart failure with preserve ejection fraction patients (Kosmala et al., 2013). Dia et al., subjected rats to LAD ligation followed by Ivabradine injection. Myocardial infarction led to a significant reduction in cardiac function indicated by an elevation in left ventricular end diastolic pressure, decreased left ventricular systolic pressure (LVSP) and reduced ascending/descending rate of left ventricular pressure (±dp/dt_max_) compared to sham rats. Cardiac dysfunction was prevented *via* Ivabradine treatment as rats demonstrated increased LVSP and ±dp/dt_max_ as well as decreased LVEFP levels. Ivabradine treatment significantly reduced the infarct size and cardiomyocyte apoptosis. Further analysis revealed that myocardial infarction-subject rats had significantly reduced expression of Beclin-1, LC3, ATG5 and ATG7, whereas p62 expression was upregulated. On the contrary, Ivabradine treatment reversed these changes. Consistently, the activated levels of PI3K, AKT, mTOR and p70S6K were increased in the myocardial infarction -subjected rat heart tissue but Ivabradine treatment inhibited these changes, thus enhancing autophagy (Dai et al., 2021). A similar effect was seen in a study by Yu et al., who investigated Ivabradine in the cardiac model of TAC. They found that Ivabradine alleviated left ventricular dysfunction, cardiac hypertrophy, fibrosis, and cardiomyocyte apoptosis. in a dose-dependent manner. Molecular analysis showed that Ivabradine modulated the PI3K/Akt/mTOR/p70S6K pathway (Yu et al., 2019).

#### 3.3.9 Tat-Beclin 1

Tat-Beclin 1 is a cell permeable peptide, derived from a region within the autophagic protein Beclin-1 and linked to the HIV-1 Tat protein to aid cell permeability. It is a portal inducer of autophagy that causes the release of endogenous Beclin-1 from negative autophagic regulatory proteins such as Golgi-associated retrograde protein 1 (GARP-1) (Shoji-Kawata et al., 2013). Various *in vivo* and *in vitro* studies have demonstrated that Tat-Beclin-1 can be used pharmacologically to promote autophagy. Xie et al., demonstrated that Tat-Beclin-1 administration in mice subjected to ischaemia/reperfusion injury resulted in improved cardiac function, reduced infarct size and decreased oxidative stress (Xie et al., 2021b). Similarly, Shirakabe et al., found that Tat-Beclin-1 treatment protected against pressure-overload stress-induced cardiac and mitochondrial dysfunction by increasing autophagic flux in the mouse heart (Shirakabe et al., 2016a). Daily Tat-Beclin 1 Treatment in neonatal and adult mice has been shown to be well tolerated. Clinical parameters including renal, hepatic and haematological function were normal in Tat-Beclin-1 treated 3 month-old mice (Shoji-Kawata et al., 2013).

#### 3.3.10 Fasting

Apart from the drug-induced autophagy, various other strategies have been proposed to induce beneficial autophagic processes for the prevention and treatment of age-associated cardiovascular diseases. One alternative method is a fasting-mimicking diet (FMD), which is composed of low calories, sugars, and protein while high in unsaturated fats. One study of 100 generally healthy participants discovered that 5-day FMD per months can safely and effectively reduce age-associated markers/factors, thus preventing diseases (Wei et al., 2017). Similarly, short-term fasting (3-day) has been proved to exert beneficial effects during cardiac ischemic/reperfusion injury in rats, as it is shown to reduce the infarct size, total number of premature ventricular complexes, the duration of ventricular tachycardia as well as improving mitochondrial redox state. These improvements may be partially due to fasting-induced mitophagy (Šnorek, 2012). Not only in the heart, mitophagy plays beneficial role in the skeletal muscle. During acute exercise, high energy demands induce proliferator-activated receptor-γ coactivator-1α (PGC-1α) to initiate mitophagy, which in essential for the mitochondrial biogenesis. The deletion of PGC-1α caused decrease in nearly half of the mitochondrial content and autophagy-related genes, thus reducing the mitochondrial performance (Vainshtein et al., 2015).

Changes in nutrients has been linked to a process known as O-GlcNAcylation (Sugahara et al., 2019). The addition of O-linked β-N-acetylglucosamine (*O*-GlcNAc) to serine/threonine residues of proteins, is a dynamic, reversible and nutrient-sensing post-translational modification (Fülöp et al., 2007). O-GlcNAcylation is catalysed and removed by O-GlcNAc transferase (OGT) and O-GlcNAcase (OGA), respectively (Rahman et al., 2020). This process regulates a plethora of cellular processes including, protein stability, protein-protein interaction, proteostasis and autophagy (Chatham et al., 2021). It is increasingly recognised that alterations in protein O-GlcNAcylation contributes to the development and progression of age-related diseases (Banerjee et al., 2016). Sustained O-GlcNAcylation was observed in diabetic and hypertrophic hearts, which was linked to cardiac dysfunction (Chatham et al., 2021). Furthermore, animal models and human patients with hypertrophic or failing hearts have also reported to have increased protein O-GlcNAcylation (Mailleux et al., 2016). In contrast, acute O-GlcNAcylation is suggested to be cardioprotective, with loss of O-GlcNAcylation increasing susceptibility to oxidative stress (Wright et al., 2017). Recently, increasing focus is on studying the relationship between O-GlcNAcylation and autophagy (Yu et al., 2020). Gelinas et al., demonstrated that the use of A769662 to activate AMPK led to decreased pathological cardiac hypertrophy in both *in vitro* and *in vivo* models by decreased protein O-GlcNAcylation (Gélinas et al., 2018). However, further research involving age-related cardiac diseases and O-GlcNAcylation is required to delineate potential therapeutic mechanisms.

## 4 Mitophagy

Mitophagy, also referred to as mitochondria autophagy, regulates the removal of unwanted and damaged mitochondria. Mitochondria are the main organelles responsible for the production of approximately 90% of all ATP in organisms therefore, serving as cellular powerhouses. They are highly abundant in the heart which is essential in providing enough energy to meet the demands of cardiac contraction and relaxation, especially during physical activity (Brown et al., 2017). Abnormal mitochondrial function has been associated with decreased exercise tolerance in heart failure patients, which not only occurs due to the reduced ability to produce ATP but also as a result of damage and death of cardiomyocytes. Under normal conditions, ROS produced in the mitochondria, as byproducts are rapidly neutralised by cellular antioxidant systems (Zorov et al., 2014). Impaired mitochondrial function not only compromises cellular respiration and ATP production but also impairs ROS production thereby increasing oxidative stress that can induce cellular damage. To remove the damaged mitochondria, a form of autophagy known as mitophagy is activated. Notably, mitophagy is particularly important for cellular homeostasis owing to the essential role played by mitochondria in the bioenergetics of the cardiovascular system. Currently, the main mitophagy pathway studied is PINK1 (PTEN-inducible kinase 1) and Parkin. Other mitophagy inducer pathways that have been documented include NIX1 (NIP3-like protein X), BNIP3 (BCL2/adenovirus E1B 19-kDa-interacting protein 3) and FUNDC1 (FUN14 Domain Containing 1) (Liu et al., 2014).

### 4.1 PINK1/Parkin Pathways

**FIGURE 2:**
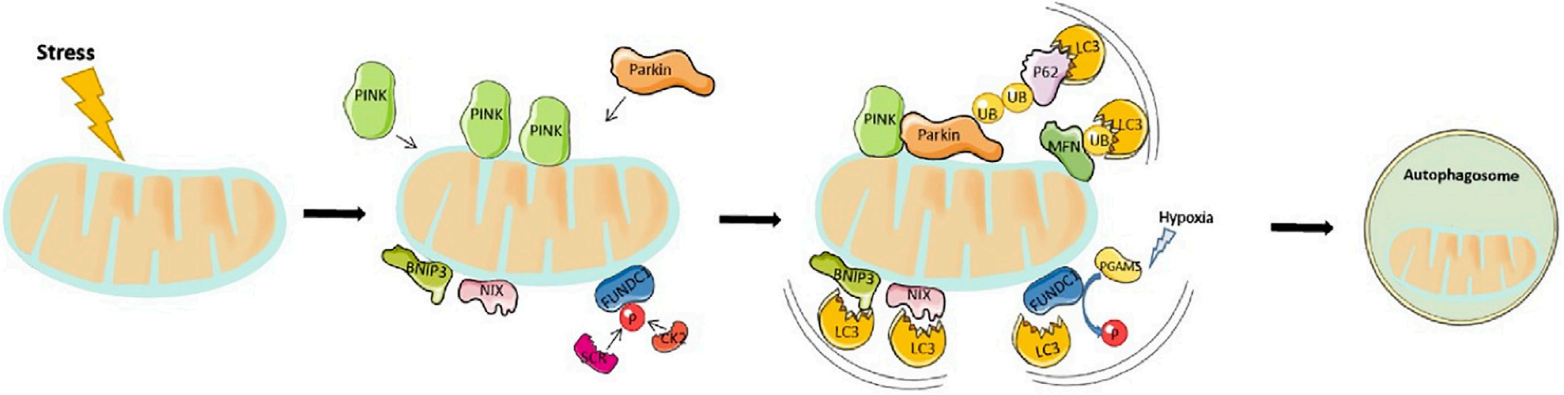
Overview of mitophagy pathway: Mitophagy is driven by either Parkin-dependent or independent pathways. Under stress, PINK1 accumulates in the outer mitochondrial membrane (OMM) and promotes Parkin recruitment. Activated Parkin, in turn, promotes ubiquitination of the outer membrane proteins. p62 recognizes phosphorylated poly-Ub chains on mitochondrial proteins and binds to LC3 to initiate the formation of autophagosome. On the other hand, the mitophagy receptors, BNIP3, NIX, and FUNDC1 localize to the OMM, and interact directly with LC3 to mediate mitochondrial degradation.

The 64-kDA protein, PINK1 has been shown to detect changes in mitochondria quality. It contains a mitochondrial targeting sequence (MTS) and under normal mitochondrial function, is transported to the inner mitochondrial matrix where it is cleaved and degraded by proteases within the mitochondria. During cardiac stress or ageing, the old and damaged mitochondria lose their membrane potential becoming more depolarised. In response, PINK1 is no longer imported to the inner membrane and instead concentrates on the outer membrane. Subsequently, it activates and recruits the cytosolic E3 ubiquitin ligase, Parkin, to the mitochondria. This is achieved *via* two mechanisms. First, PINK1 directly phosphorylates Parkin at Ser65 resulting in a conformational change and Parkin activation. Second, the Ser65 site on Parkin is homologous to the phosphorylation site on ubiquitin, therefore PINK1 phosphorylates ubiquitin which subsequently competes with the autoinhibitory domain within Parkin to stabilize and activate it (Ge et al., 2020). Notably, Parkin-mediated ubiquitination generates two main effects. Firstly, it helps p62 to link ubiquitinated proteins to LC3 on the mitochondria, which are subsequently degraded by lysosomes. Second, several proteins located on the mitochondria surface undergo Parkin-mediated ubiquitination. These include mitofusin 1 and mitofusin 2, both of which modulate mitochondrial fusion. This proteasome-mediated breakdown of ubiquitinated mitofusins further promotes mitochondrial fragmentation and mitophagy (Kazlauskaite et al., 2014). A summary of this processes can be seen in Figure 2.

### 4.2 The BNIP3/NIX Pathways

BNIP3 and NIX are apoptotic proteins that promote the opening of mitochondrial permeability transition pore (mPTP), and activation of Bax/Bak to enhance mitochondrial membrane permeability and induce apoptosis. Structurally, BNIP3/NIX has an LC3-interacting region (LIR) motif that directly interacts with LC3 on autophagosomes, thereby defining NIX/BNIP3L as a mitophagy receptor. The interaction between BNIP3 and LC3 requires their homodimerization. Notably, NIX, together with some core Atg proteins (Nishida et al., 2009; Novak et al., 2010) mediates mitophagy that occurs during differentiation of mammalian red blood cells or reticulocytes, while BNIP3 modulate the removal of endoplasmic reticulum and mitochondria under hypoxic conditions (Hanna et al., 2012). It can be speculated that hypoxia activates these mitophagy receptors. An important mechanism is that HIF or FOXO3 regulate the transcription of BNIP3/NIX (Cao et al., 2013). Previous studies have also demonstrated that phosphorylated BNIP3, at Ser17 and Ser24, triggers its interaction with GATE-16 and LC3-B, thereby promoting the occurrence of mitophagy (Zhu et al., 2013). Additional evidence has demonstrated that NIX/BNIP3L interacts with Rheb thereby stimulating mitophagy in cells with a high level of oxidative phosphorylation (Melser et al., 2013).

### 4.3 FUNDC1 Pathways

FUNDC1 is a protein found in the outer mitochondrial membrane that possesses an LIR region allowing LC3 interaction. Under basal conditions, the LIR motif is inhibited *via* phosphorylation of Tyr18 and Ser13 by Src kinase and CK2, respectively. These two constitutively active kinases are highly abundant, a phenomenon that ensures inhibition of mitophagy. However, under acute pathological conditions, the LIR region is dephosphorylated allowing, FUNDC1 to bind to LC3 II to mediate mitochondrial autophagy. Previous studies have shown that FUNDC1 Ser17 is phosphorylated by ULK1 to enhance mitophagy. In addition, FUNDC1 binds with optic atrophy-1 (OPA1) or dynamic related protein-1 (Drp1) to regulate mitochondrial fusion or fission. Under normal circumstances, FUNDC1 interacts with OPA1 to promote mitochondrial fusion, while under mitochondrial stress, they dissociate. At the same time, they recruit DRP1 in the cytoplasm to mitochondria, thereby promoting mitochondrial fission and mitophagy (Wu et al., 2014).

### 4.4 Role of Mitophagy in Ageing

Mitophagy employs three signalling pathways under different physiological and pathophysiological conditions, which interact with each other. To date, however, the specific relationship among the three pathways remains unclear, necessitating further explorations. Results from a previous study showed that NIX overexpression promotes Parkin translocation to mitochondria, while NIX-mediated mitophagy is reduced in Parkin gene knockout mice (Lee et al., 2011).

Mitophagy regulates both mitochondrial quantity and quality. Notably, ageing hearts generally exhibit low mitophagy levels, which can lead to dysfunctional mitochondrial accumulation, further increasing ROS generation and exacerbating cardiomyocyte injury and dysfunction (Shirakabe et al., 2016b). It has been reported that AMPK and Akt deficiency predisposes mice to cardiac ageing most likely due to comprised mitophagy. The study also unmasked the age-related decline in mitophagy components including, FUNDC1, BNIP3, PINK1, Parkin and lysosome biogenesis factor TFEB (Transcription Factor EB) (Wang et al., 2019b).

Additional evidence showed that ageing caused cardiac dysfunction in mice, that was associated with decreased levels of mitophagy. Parkin overexpression were found to ameliorate heart failure symptoms (Hoshino et al., 2013). Previous studies have shown that silencing of the PINK1 gene impairs mitochondrial function in cardiomyocytes, and subsequently elevates levels of oxidative stress, thereby causing heart failure. Parkin is dispensable under normal conditions, however, following stress, Parkin deficiency leads to the accumulation of dysregulated mitochondria in cardiomyocytes (Kubli et al., 2013). In addition, PINK1-KO mice developed left ventricular dysfunction and cardiac hypertrophy, during earlier stages of life, while PINK1 expression was significantly downregulated in the left ventricles of patients with end-stage heart failure (Billia et al., 2011). Furthermore, Nix/BNip3 double cardiac knockout mice rapidly developed heart failure, after TAC, while knocking out NIX alone suppressed myocardial cell apoptosis and improved myocardial contractility (Dorn, 2010). Interestingly, cardiac-specific overexpression of NIX caused activation of apoptosis in myocytes, and subsequent development of heart failure (Yussman et al., 2002). Conversely, excessive mitophagy can cause a massive mitochondria loss, consequently compromising the energy supply of cardiomyocytes. Therefore, maintaining a mitophagy balance is imperative to healthy cardiomyocyte function (Liang and Gustafsson, 2020).

### 4.5 Mitophagy Targeting Drugs

The involvement of dysregulated mitophagy in the development of CVDs has been widely reported. Targeting mitophagy by pharmacological manipulation to prevent or treat CVD may be an effective strategy. Compared to autophagy, much less comparable information is available in relation to specific targeting of mitophagy as a therapeutic avenue in CVDs.

#### 4.5.1 Simvastatin

Statins are 3-hydroxy-3-methylglutaryl coenzyme A (HMG-CoA) reductase inhibitors that limit cholesterol biosynthesis. Although the role of statins in inducing autophagy in various cell lines has been well documented, limited research has investigated their role in the setting of cardiomyocytes, both *in vivo* and *in vitro.* To address this, a recent study employed the mouse model of ischaemia/reperfusion injury. It was demonstrated that Simvastatin suppressed mTOR signalling and activated Parkin-dependent mitophagy to confer cardioprotection. Simvastatin increased Parkin and p62/SQSTMI translation to increase mitophagy and migrated the ischaemia/reperfusion injury induced damage to the cardiac mitochondria. However, whether other statin drugs confer cardioprotection by inducing mitophagy remain to be investigated (Andres et al., 2014).

#### 4.5.2 Natural Compounds

Plant-derived polyphenols have widely been shown to confer numerous health benefits. Research is now discovering their role in modulating mitophagy. Following ischaemia/reperfusion injury, treatment with the polyphenolic antioxidant Resveratrol was shown to upregulate SIRT1 which cooperates with SIRT3 to elevate FOXO3 levels. This subsequently activated PINK1/Parkin signalling resulting in mitophagy activation. Resveratrol can also enhance mitochondrial elongation, modulate Drp1 expression and increase LC3-II to degrade damaged and dysfunctional mitochondria in aged cardiomycoytes (Das et al., 2014).

Quercetin is a flavonoid compound that possesses various pharmacological effects including coronary artery dilation, increased blood flow and capillary resistance. Both *in vivo* and *in vitro* experiments have shown that following cardiac stress such as ischaemia/reperfusion injury, Quercetin can significant improve mitochondrial function and energy metabolism to limit damage to the cardiac tissue (Wang et al., 2013). Similar to Resveratrol, Quercetin administration leads to a FOXO3-mediated increase in mitophagy, however, this is achieved *via* an ERK2 and AMPK dependent pathway (Tan and Wong, 2017).

Dimeric xanthones, Gerontoxanthone I and Macluraxanthone can increase the cellular accumulation of Parkin puncta and promote mitophagy. In a prior study, the protective role of Gerontoxanthone and Macluraxanthone against ischaemia/reperfusion injury was demonstrated in H9C2 cell lines. The authors found that both drugs stabilized PINK1 on the mitochondrial outer membrane and increased the recruitment of Parkin to the mitochondria. These effects were abolished in Parkin knockdown cells indicating that Gerontoxanthone and Macluraxanthone upregulate mitophagy *via* PINK1-Parkin dependent manner. However, if this benefit can be replicated *in vivo* require further investigation (Xiang et al., 2020).

Another promising mitophagy inducer is Urolithin A, a gut microflora-derived metabolite. Urolithin A administration in a mouse model of type II diabetes demonstrated its ability to induce mitophagy in cardiac fibroblast to limit the extent of cardiac dysfunction and fibrosis. This was achieved through increased Drp1 expression whereas PINK1 and phosphorylated Parkin remain unchanged indicating non-canonical mitophagy regulation (Wu et al., 2019a). *In vivo* Urolithin A supplementation also attenuated excessive inflammation in the cardiac tissue following hyperglycaemia (Savi et al., 2017). Furthermore, a phase 1 clinical trial has demonstrated the safety of Urolithin A administration (Andreux et al., 2019). Together, these studies suggest Urolithin A could induce mitophagy to induce cardioprotection following stress.

#### 4.5.3 Nicotinamide Adenine Dinucleotide

Nicotinamide adenine dinucleotide (NAD^+^) is a fundamental molecule that is a mitophagy inducer. However, the level of NAD^+^ expression decreases in an age-dependent manner and also during cardiac disease, so targeting NAD^+^ restoration could provide therapeutic benefits. Supplementation with NAD^+^ precursors upregulated the expression of SIRT1, thus providing protection against aging (Verdin, 2015). Studies have utilized Nicotinamide riboside to increase NAD^+^ levels and have shown multiple cardiac protective benefits including improved cardiac function in mice with TAC-induced dilated cardiomyopathy (Diguet et al., 2018). Several human clinical trials have demonstrated the bioavailability and safety of Nicotinamide riboside (Mehmel et al., 2020). Other studies have used Nicotinamide mononucleotide to increase NAD^+^ levels. In the heart, supplementation of nicotinamide mononucleotide, a product of Nampt in the NAD+ salvage pathway, protected hearts from ischaemia/reperfusion stress through the activation of SIRT1 (Yamamoto et al., 2014). Other studies have shown that Nicotinamide mononucleotide supplementation can normalize NAD^+^ levels and maintain SIRT3 activity, resulting in increased mitophagy and improved mitochondrial function in heart failure mice (Karamanlidis et al., 2013). NAD^+^ mediated sirtuin activation can also inhibit mTOR to stimulate autophagy and increase mitophagy by increasing the expression of Parkin, PINK1 and BNIP3 (Aman et al., 2021). NAD+ supplementation has also been linked to regulating O-GlcNAcylation. Recently, Zou et al., demonstrated that NAD+ was capable of blocking glucose deprivation induced increase in O-GlcNAcylation (Zou et al., 2015). However, the exact signalling mechanism of NAD+ in other cardiac diseases well as the safety of NAD^+^ supplement needs to be further elucidated.

#### 4.5.4 UMI-77

Surprisingly, a recent study found that UMI-77, a BH3-mimetic for MCL-1 and can modulate mitophagy. MCL-1 is a mitophagy receptor that can directly interact with LC3 to induce mitophagy. UMI-77 was able to enhance MCL-1 and LC3 interaction to selectively degrade damaged mitochondria (Cen et al., 2020). Although the study investigated the therapeutic significance of UMI-77 in Alzheimer’s disease, it has important implications for CVD management. Studies have shown that deletion of MCL-1 in adult cardiomyocytes leads to accumulation of dysfunctional mitochondria due to defective PINK1/Parkin signalling (Cen et al., 2021). Therefore, further research investigating MCL-1 and UMI-77 in cardiac models is warranted.

#### 4.5.5 TEMPOL

TEMPOL (4-hydroxy-2,2,6,6-tetramethylpiperidin-1-oxyl) has been shown to reduce ROS levels and counteract the age-related decline in mitophagy (Morciano et al., 2020). It was found that pretreatment with a TEMPOL, a potent antioxidant, increased PINK1 expression and Parkin accumulation in mitochondria, as well as elevating mitochondrial ubiquitination following ischaemia reperfusion injury and in aged cardiomyocytes (Ma et al., 2017).

## 5 Endoplasmic Reticulum Stress Response

The endoplasmic reticulum (ER) is a large specialised organelle that coordinates the folding and translocation of secretory and transmembrane proteins, biosynthesises lipids and sterols, and the signalling, uptake, and storage of intracellular Ca^2+.^ Under basal conditions, ER homeostasis is maintained as the equilibrium between protein load and folding capacity is sustained. However, during either physiological or pathological stress, an imbalance can occur due to various reasons such as accumulation of misfolded proteins and increased protein synthesis, thus causing ER stress. Factors such as mutated proteins, dysregulated disulphide bond formation, and impaired protein glycosylation can further contribute to ER stress (Ren et al., 2021a).

**FIGURE 3:**
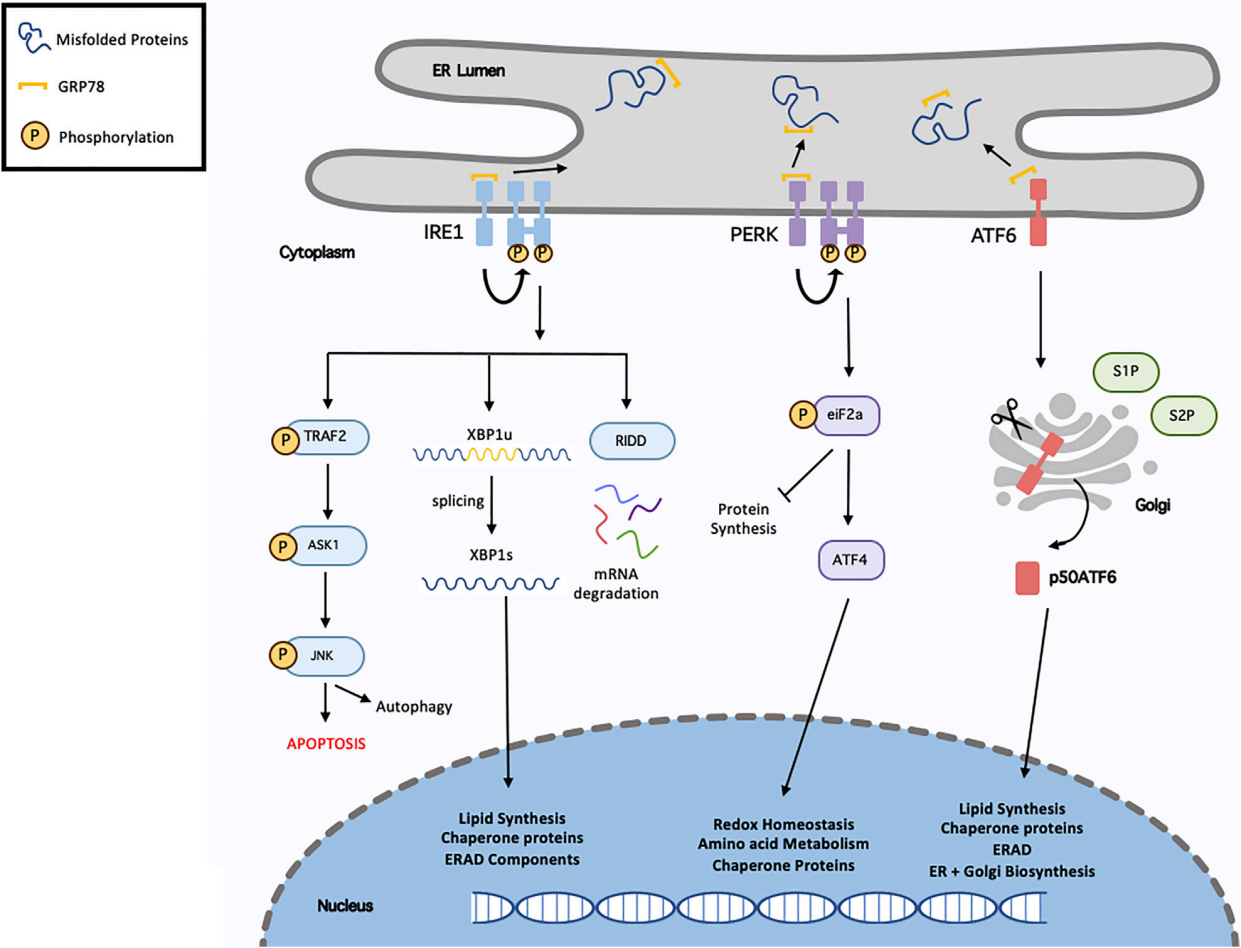
Overview of unfolded protein response. Following endoplasmic reticulum (ER), misfolded/unfolded proteins bind and sequester GRP78 (Binding immunoglobulin protein), thus activating the unfolded protein response (UPR). This results in activation of the three signalling cascades, IRE1 (inositol requiring enzyme 1), PERK (protein kinase RNA-like ER kinase) and ATF6 (activating transcription factor-6). Following dissociation from GRP78, IRE1 undergoes homodimersation and autophosphorylation. It can then induces XBP1 (x-box binding protein 1) splicing, activated RIDD (regulated IRE1-dependent decay) or recruits TRAF2 (TNF receptor associated factor 2) resulting in ASK1 (apoptosis signal-regulating kinase 1) phosphorylation, which then phosphorylates the c-Jun N-terminal kinase (JNK). Activation of the PERK pathway, results in eIF2α (eukaryotic translation initiation factor 2α) phosphorylation, which in turn inhibit protein synthesis and activate ATF4 (activated transcription factor 4). In terms of ATF6, it translocates to the Golgi where it is cleaved and migrates to the nucleus. XBP1, ATF4 and ATF6 regulate various genes called in chaperone proteins synthesis, ER-associated degradation (ERAD), lipid synthesis and redox homeostasis.

To counteract ER stress following either physiological or pathological stimuli, a number of adaptive mechanisms are activated, including the unfolded protein response (UPR) and the ER-associated degradation (ERAD) pathway. Originally, the UPR aims to alleviate ER stress thus restoring homeostasis. This can be achieved by increasing the degradation of misfolded proteins and inhibiting protein translation to decrease protein load. However, severe or prolonged stress initiates the ERAD pathway to induce cell death (Ozcan and Tabas, 2012). The UPR consists of a set of transmembrane ER-resident proteins, including, inositol-requiring protein 1 (IRE1), PKR-like endoplasmic reticulum kinase (PERK), and activating transcription factor 6 (ATF6). Under basal conditions, the chaperone glucose regulatory protein 78 (GRP78, also known as BiP) is bound to these proteins preventing their activation. Following stress, GRP78 dissociates allowing the activation of these transmembrane proteins (Ren et al., 2021a). A summary of this processes can be seen in Figure 3.

IRE1 is the most evolutionary conserved protein amongst UPR transducers, possessing both endoribonuclease and kinase activity. Upon activation, IRE1 excises a 26-nucleotide intron from X-Box Binding Protein 1 (XBP1) mRNA, resulting in the transcriptionally active spliced-XBP1 (XBP1s). Once activated, XBP1s is a potent transcription factor that regulates genes involved in protein quality control and ERAD. There are two main IRE1 isoforms, IRE1α and IRE1β. IRE1α has stronger XBP1 splicing activity whereas IRE1β can act as a negative-suppressor of IRE1α (Ren et al., 2021a). IRE1 is also involved in the IRE1-dependent decay (RIDD) pathway. As IRE1 possesses RNAse activity it is capable of degrading mRNA and miRNAs localised in the ER membrane thereby reducing protein influx into the ER lumen (Coelho and Domingos, 2014).

PERK is a protein kinase consisting of both kinase and cytoplasmic domains. PERK undergoes trans-autophosphorylation to activate its cytosolic kinase domain, which facilities the phosphorylation of eukaryotic initiation factor 2 (eIF2a). This ultimately leads to attenuation of protein translation, reduced flow of newly synthesised proteins into the ER, and permit the cell to recover from the misfolded protein load and maintain protein homeostasis. Phosphorylated eIF2a also increases the translation of Activating Transpiration Factor 4 (ATF4) mRNA to increase the expression of various UPR genes (Liu et al., 2015).

Upon release from GRP78, ATF6 migrates to the Golgi apparatus where it is cleaved by site-1 and site-2 proteases. The 50 kDa N-terminal fragment, ATG6α, migrates to the nucleus where it upregulates several genes involved in the UPR and ERAD pathway to aid in protein folding and lipid biogenesis. The other ATF6 fragment, ATF6β is suggested to inhibit ATG6α activity and possess less transcriptional activity. The physiological role of ATF6β during basal conditions and stress is significantly less documented as opposed to ATF6α (Hillary and Fitzgerald, 2018).

ERAD pathway functions to transport misfolded proteins out of the ER to allow degradation by the ubiquitin proteasome system (UPS). The UPS plays a vital role in maintaining protein quality control by selectively degrading proteins, in a process called ubiquitination. This involves the addition of a 76-amino-acid polypeptide to specific lysine residues on the target protein. This is achieved by a three-step cascade involving ubiquitin-activating (E1), ubiquitin-conjugating (E2) and ubiquitin ligase (E3). During the reaction, a bond is formed between the COOH group of the C-terminal of ubiquitin is formed and NH_2_ of lysine residue. The majority of proteins require the addition of a poly-ubiquitin chain for destruction. To achieve this, the ubiquitin molecule contains seven lysine residues, which can form a polymeric ubiquitin chain by sequentially adding ubiquitin moieties (Berner et al., 2018). Poly-ubiquitinated protein degradation is catalysed by the ATP-dependent multi-catalytic complex, 26S proteasome. This complex comprises two assemblies, the 20S core particle and the 19S regulatory complex. The 20S proteasome forms a barrel-shape structure composed of 28 units containing two peripheral α subunits, regulating entry of unfolded proteins and two β subunits harbouring proteolytic activity. The 19S cap complex contains six AAA ATPases (Rpt1-Rpt6) which form the RpT ring. This ring is responsible for unfolding incoming proteins in an ATP-dependent manner and transporting the unfolded protein chain into the cylinder for degradation. Other non-ATPase protein are present in the 19S cap, including Rpn10 and Rpn13 that function as ubiquitin-receptors (Kocaturk and Gozuacik, 2018). Following proteasomal degradation, the resulting small peptides are further broken down by cytosolic peptidases producing amino acids that can be recycled (Voges et al., 1999).

Several key chaperone proteins are present in the ER to aid in the identification and folding of misfolded proteins. Such proteins include calreticulin, calnexin, protein disulphide isomerase (PDI), as well as glucose-regulated protein 94 (GRP94) (Brown and Naidoo, 2012). Numerous studies have demonstrated the involvement of the UPR and ERAD signalling pathways in different CVD models, including, myocardial infarction (Belmont et al., 2010), diabetes mellitus (Wu et al., 2021), hypertension (Wang et al., 2014a), pressure overload and heart failure (Doroudgar et al., 2015).

### 5.1 Molecular Pathways Underlying Reduced Endoplasmic Reticulum Stress Response in Cardiac Ageing

Studies have shown that the function of the UPR in ageing and different diseases is complex. Multiple factors influence the ER response including the nature, timing and strength of the specific stress insult (Lindholm et al., 2017). During acute stress insults, the ER is capable of clearing accumulated unfolded/misfolded proteins and aggregates *via* rapid activation of the UPR to maintain cellular homeostasis. In contrast, during prolonged chronic stress the protein load exceeds the ER folding capacity and the UPR fails to restore cellular homeostasis leading to activation of apoptotic signalling (Liu and Connor, 2015). The effect of ageing on proteostasis has been investigated extensively in various tissues. Numerous studies have demonstrated that ER stress is a hallmark feature in the pathogenesis of several age-related CVDs including type II diabetes and heart failure (Yang et al., 2020). During ageing, there is a progressive decline in the UPR function and an increase in pro-apoptotic signalling in several tissues, such as the liver, lung, brain, kidney, and heart. The total number of UPR pathway components including, PDI, GRP78, GRP94, and calnexin decline with age. Furthermore, increased oxidisation leads to structural changes within chaperone proteins which significantly attenuates their function and comprises the ER response (Li et al., 2019b). During ageing, the increased level of misfolded and aggregated proteins is a particular burden in long-lived, non-dividing cells such as cardiomyocytes. Furthermore, increased ER damage and reduced UPR signalling significantly attenuate the ability of cells to remove protein aggregates (Hipp et al., 2019). Together these findings support the notion that therapeutically targeting ER stress and the UPR may be a viable approach to targeting ageing and disease.

### 5.2 Drugs Targeting Endoplasmic Reticulum Stress Response

There is clear evidence linking the loss of ER-mediated proteostasis and ageing-related CVDs. Animal and cellular models using gain or loss of function of UPR proteins have demonstrated the therapeutic potential of targeting the ER. Therefore, scientific attention has turned to finding novel pharmacological therapies to target impaired proteostasis and maintain cardiac function during disease and ageing. A summary of the autophagy activators and their limitations can be seen in Table 2.

**TABLE 2:** List of drugs shown to modulate unfolded protein response.

Drug name	Mode of action	Research progression	Major limitations	References
Empagliflozin	↓ CHOP, ↓ Caspase-12	FDA approved for the treatment of type II diabetes mellitus	Unknown mechanism	Zhou and Wu, (2017)
		In clinical trial for treatment of cardiovascular disease		
B7-33	↓ CHOP, ↓ GRP78	Currently only used experimentally	Unknown mechanism	Devarakonda et al. (2020)
Icariside II	↓ CHOP, ↓ GRP78, ↓ PERK, ↓ ATF4, ↓ mTORC1	In clinical trial for investigation of cardiovascular health	Limited information regarding pharmacokinetic and metabolism	Wu et al. (2019b)
Trigonelline	↓ eIF2a, ↓ GRP78, ↓ GRP94, ↓ Caspase-3, ↓ Caspase-9, ↓ GRP78, ↑ Bcl-2, ↑ Bcl-XL	In clinical trial for investigation of cardiovascular health	Poor bioavailability and Solubility	Tharaheswari et al. (2014)
Notoginsenoside R1	↓ Caspase-12, ↓ CHOP, ↓ p-JNK, ↓ p-PERK, ↓ ATF6, ↓ IRE1a	Currently only used experimentally	Poor bioavailability and solubility	Yu et al. (2016)
Araloside C	↓ ATF6, ↓ PERK/eIF2a	Currently only used experimentally	Poor bioavailability and Solubility	Du et al. (2018)
Propranolol and Metoprolol	↓ XBP-1, ↓ GRP78,↓ CamKII	FDA Approved for use as a beta-blocker	Unknown mechanism	Ni et al. (2011)
Salubrinal	↑ P-eIF2a, ↓ CHOP, ↓ Caspase-12	Currently only used experimental	Unknown mechanism	Rani et al. (2017b)
Elatoside C	↓ GRP78, ↓ CHOP, ↓ Caspase-12	Currently only used experimental	Poor bioavailability and solubility	Wang et al. (2014b)
Metformin	↑ PERK/ATF4	FDA Approved for treatment of type II diabetes mmellitus	Non-selective	Quentin et al. (2012)
4-PBA	Binds to misfolded proteins	FDA approved for treatment of urea cycle disorder	Non-specific as it can modulates with multiple cellular processes	Jian et al. (2016b)
				Park et al. (2012)
TUDCA	Binds to misfolded proteins	In clinical trials for treatment of ulcerative colitis and transthyretin Amyloid cardiomyopathy	Unknown safety following long-term administration	Rani et al. (2017a)
				Radwan et al. (2020)
Apelin-13	↓ JNK, ↓ CHOP, ↓ Caspase-12	In clinical trial for treatment of chronic kidney disease	Poor stability *in vivo*	Samura et al. (2016)
Berberine	↓ PERK, ↓ eIF2a, ↓ CHOP, ↓ ATF4, ↓ GRP78, ↓ Caspase-12	In preclinical development	Poor bioavailability and solubility	Zhao et al. (2016)
			Unknown mechanism adverse gastrointestinal effect	Liao et al. (2018)
			—	Yin et al. (2008)
Olmesartan	↓ Caspase-12, ↓ GRP78, ↓ p-JNK	FDA approved for treatment of hypertension	Unknown mechanism	Sukumaran et al. (2011)
Atorvastatin	↓ GRP78, ↓ CHOP, ↓ Caspase-12	FDA approved for treatment of high cholesterol	Non-specific	Song et al. (2011)

#### 5.2.1 Empagliflozin

SGLT-2 inhibitors have also been associated with reduced ER stress during cardiac stress. Empagliflozin has been shown to protect against ER stress-induced cardiomyocyte apoptosis. Zhou and Wu 2017 used STZ-induced diabetic rats treated with Empagliflozin to examine the impact it has on ER stress in diabetic cardiomyopathy. They found that Empagliflozin treatment led to a significant reduction in the expression of CHOP and caspase 12 activation, suggesting reduced ER stress. They also found that the mRNA expression of XBP1, ATF4, and TRAF2 was decreased (Zhou and Wu, 2017). Despite the growing evidence stating the cardioprotective effects of SGLT-2 inhibitors, more research is needed to investigate the role of these drugs on different forms of CVD. It is also currently unclear if the protective effects of SGLT-2 inhibitors on the heart are due to a systemic effect or a direct effect on the myocardium. SGLT-2 is predominately found in the proximal tubules in the kidney and does not exist in the myocardium, however, Park et al., showed that Porcine coronary artery endothelial cells were able to express SGLT-2 upon exposure to glucose despite having no basal SGLT-2 expression (Park, 2018).

#### 5.2.2 Glucagon-1 Like Peptide Agonist

Glucagon-1 like peptide (GLP-1) is a member of the pro-glucagon incretin family that acts *via* the GLP-1 receptor. Recent studies have demonstrated that activation of GLP-1 can reduce ER stress. Liraglutide is a GLP-1 receptor agonist that can activate the GLP-1 pathway. Liraglutide was shown to alleviate ER stress in endothelial cells isolated from diabetic patients. Acute treatment with Liraglutide decreased the expression of phosphorylated IRE1α and JNK whereas insulin-stimulated endothelial nitric oxide synthase activation was restored (Bretón-Romero, 2018). A further study found that Liraglutide was capable of improving cardiac function in rats subjected to diabetic cardiomyopathy. Mechanistically, Liraglutide attenuated ER stress by decreasing IRE1α/sXBP1 signalling and CHOP-mediated cell death (Ji et al., 2014). Another GLP-1 agonist, Exendin-4 has been linked to modulating ER stress in the diabetic myocardium. It has been shown that Exendin-4 treatment can prevent thapsigargin and hydrogen-peroxide induced cardiomyocyte death. Cardiomyocytes exhibited reduced ER stress evident by decreased GRP78 and CHOP expression (Younce et al., 2013). A plethora of scientific studies have investigated the role of GLP-1 in diabetes, but limited research focused on its role in other CVDs. This is further hindered by the finding that although Dulaglutide (GLP-1 agonist) was capable of lowing blood glucose levels, systolic blood pressure and body weight in type II diabetes patients, no significant difference in cumulative incidence of cardiovascular outcomes was seen. This indicates that GLP-1 may only be capable of reducing ER stress in diabetes (Gerstein et al., 2019). This warrants further research regarding the role of GLP-1 in CVDs.

#### 5.2.3 Natural Compounds

Recently, several natural compounds have gained attention due to their ability to modulate protein homeostasis pathways and to exert a beneficial role during both ageing and CVDs. The phytochemical compound present in fenugreek seeds, Trigonelline, was identified to possess anti-apoptotic properties. Following palmitic stress-induced ER stress, Trigonelline reduced eIF2a phosphorylation and expression of GRP78/94, attenuating metabolic stress (Khound et al., 2018). *In vitro* experiments utilising H9C2 cells subjected to hydrogen-peroxide induced apoptosis revealed that Trigonelline downregulated the expression of proapoptotic caspases such as caspase-3 and caspase-9. Instead, anti-apoptotic proteins Bcl-2 and Bcl-XL expression were upregulated (Ilavenil et al., 2015). However, before this compound can be applied therapeutically to treat CVDs, further research is needed as some reports have suggested that caspase inhibitors can induce necrosis (Amen et al., 2019).

Icariside II, a PDE-5 inhibitor, is a flavonoid originating from *Epimedium brevicornum*. In traditional Chinese medicine, it has been used to treat various disorders including dementia and osteoporosis (Liu et al., 2018). Using a spontaneous hypertensive rat (SHR) model, Wu et al., demonstrated the role of Icariside II in the ER stress response pathway. The rats were intra-gastrically administrated with Icariside II up until they reached 26-weeks old before ER pathway markers were analysed. The study found Icariside II alleviated hypertension-mediated cardiomyocyte apoptosis and prevented the decline in cardiac function in the rats. Icariside II reduced the expression of GRP78, CHOP, PERK, and ATF4 at both the protein and mRNA level to alleviate the extent of ER stress, reducing the percentage of apoptotic cells (Wu et al., 2019b). Icariside II has also been implemented in autophagy. Liu et al., investigated the effect of Icariside II on cardiac hypertrophy. Mice were subjected to TAC surgery before being divided into vehicle or Icariside II groups for 6 weeks. Icariside II alleviated pressure-overload induced cardiac dysfunction, hypertrophic growth, and fibrosis. Upon examination of the underlying signalling mechanisms, it was revealed that pressure-overload stress inactivated AMPKα and activated Akt. Icariside II blunted these changes and also inhibited the mTORC1/p70s6K/4EBP1 pathway (Liu et al., 2018).

Notoginsenoside R1 is a novel saponin derived from *P.notoginseng* that has previously been shown to encompass anti-inflammatory, anti-apoptotic, and anti-oxidative ability (Xia et al., 2015). Pre-conditioning H9C2 cells or perfusing isolated rat hearts with Notoginsenoside R1 before exposing them to hypoxia/reoxygenation or ischaemia/reperfusion respectively, revealed that Notoginsenoside R1 inhibited the expression of Caspase-12, CHOP, and phosphorylated JNK. Following ischaemia/reperfusion injury the expression of various components of the ER pathway including, phosphorylated PERK, ATF6, and IRE1a increased. However, this was attenuated *via* Notoginsenoside R1 pre-conditioning. Furthermore, Notoginsenoside R1 is capable of scavenging free radicals, suppressing the extent of oxidative stress (Yu et al., 2016).

#### 5.2.4 Small Molecules

Several small molecules have been identified to regulate the UPR signalling pathway. Araloside C is a triterpenoid compound that was shown to prevent cardiomyocyte apoptosis and increase cell viability through suppression of ATF6 and PERK/eIF2a activation (Du et al., 2018). Propranolol and Metoprolol are β-adrenergic receptor blockers that can improve cardiac function in heart failure patients. It has been reported that the underlying mechanism of these drugs may be associated with regulating the ER stress response. Ni et al., subjected rats to either aortic constriction or isoproterenol injection to induce cardiac hypertrophy and heart failure. They found that Propranolol and Metoprolol treatment suppressed ER stress by decreasing the expression of CHOP resulting in decreased ER stress mediated cardiomyocyte apoptosis (Ni et al., 2011). Similarly, Li et al., demonstrated that Salubrinal treatment following myocardial infarction results in a smaller infarct size and reduced cardiomyocyte apoptosis. Mechanistically, Salubrinal increased eIF2a phosphorylation and downregulated CHOP and Caspase-12 expression (Li et al., 2015). Furthermore, the triterpenoid compound, Elatoside C demonstrated cardioprotective ability following ischaemia-reperfusion injury by increased STAT3 phosphorylation and Bcl-2 expression as well as decreased expression of ER stress proteins, GRP78, Caspase-12 and CHOP (Cheang et al., 2014).

#### 5.2.5 B7-33

Serelaxin, a novel vaso-protective drug, has recently gained considerable scientific attention as a new vaso-protective drug displaying potential therapeutic effects in heart failure. However, Serelaxin production is both costly, lengthy, and labour intensive. To overcome these issues, scientists have developed B7-33, a single-chain peptidomimetic (Marshall et al., 2017). Using both *in vivo* and *in vitro* experiments, Devarakonda et al., investigated the biological role of B7-33 during cardiac ischaemia-reperfusion injury. Adult CD1 mice were subjected to LAD ligation for 30 min followed by induction of B7-33 treatment and reperfusion for 24 h or 7 days. Echocardiological analysis revealed that B7-33 treatment maintained cardiac function reflected by the preserved fractional shortening after both 24 h and 7 days reperfusion and the lower sLVID. B7-33 treated mice also demonstrated a reduced infarct size compared to the vehicle control mice. mRNA analysis from vehicle cardiac tissue exposed to 24 h ischaemia/reperfusion injury showed increased CHOP expression, however, this increase was attenuated in B7-33 treated mice. *In vitro* experiments revealed that treatment of primary cardiomyocytes with B7-33 followed by either tunicamycin or simulated ischaemia reoxygenation exposure led to decreased GRP78 expression in an ERK1/2 dependent manner, indicating UPR pathway activation (Devarakonda et al., 2020). However, the exact signalling cascade remains to be identified. Nevertheless, B7-33 demonstrated the potential as a novel therapeutic strategy for ischaemia reperfusion injury.

#### 5.2.6 Chemical Chaperones

Chemical chaperones are able to reduce the extent of misfolded protein accumulation and aggregation by acting as endogenous chaperones to increase mutant protein trafficking and strengthen the ER folding ability (Jung and Choi, 2016). Key chemical chaperons include, 4-phenylbutyric acid (4-PBA) and Tauroursodeoxycholic acid (TUDCA). 4-PBA is an FDA-approved drug for the treatment of urea cycle disorders (Jian et al., 2016a), however, it has also shown potential therapeutic effects in various CVDs by attenuating ER stress. The low-molecular weight aromatic fatty acid, 4-PBA, modulates numerous ER components including, CHOP, GRP78, PERK, XBP1 and JNK to reduce ER stress. The cardioprotective role of 4-PBA was been shown in various diseases including cardiac ischaemia-reperfusion injury (Jian et al., 2016b), pressure-overload stress and fibrosis (Luo et al., 2015). It interacts with the hydrophobic region within misfolded proteins, preventing aggregation (Rellmann et al., 2019). In addition, TUDCA administration has been shown to reduce the expression of phosphorylated eIF2a, phosphorylated PERK and GRP78 following pressure-overload cardiac remodelling (Rani et al., 2017a). Similarly, Choi et al., demonstrated that compared to wildtype rats, spontaneously hypertensive rats had increased expression, ATF6, IRE1, XBP1, PERK, eIF2a, CHOP and GRP78. However, following TUDCA treatment there was a reduction in the expression of these ER markers. Cardiac function and systolic blood pressure were also improved with TUDCA treatment (Choi et al., 2016).

## 6 Protein Quality Control System Crosstalk

Although ER stress and autophagy involving various signalling molecules, together the different protein quality control systems act as an integrated stress response. For example, all three branches of the UPR can activate autophagy through increasing the expression of multiple ATG genes, modulating numerous kinases such as mTORC1 or AMPK or decreasing the expression of several autophagy inhibitors (Ren et al., 2021b). IRE1 and PERK branches of the UPR have been implemented in inducing autophagy by upregulating autophagy genes and increasing Beclin-1 expression (Verfaillie et al., 2010). Overexpression of XBP1s activated autophagic signaling demonstrated by enhanced LC3-II conversion and increased expression of Beclin-1 (Margariti et al., 2013). ATF6 has also been implemented in the induction of autophagy *via* Death-associated kinase 1 (DAPK1) signalling. Knockdown of ATF6 resulted in a strong reduction in DAPK1 expression, decreased Beclin-1 phosphorylation and decreased autophagosome formation (Zalckvar et al., 2009). Moreover, calcium released from the ER can stimulate Ca^2+^/calmodulin-dependent kinase kinase β (CaCMKKβ) which in turn phosphorylates and activated AMPK leading to mTORC1 inhibition and autophagy induction (Verfaillie et al., 2010). Ca^2+^ presence in the cytosol also activates DAPK1 signalling, to increase Beclin-1 and Bcl-2 phosphorylation to trigger autophagy (Zalckvar et al., 2009). Although clear links been autophagy and UPR have been acknowledged, fewer studies have explored them in the context of cardiac ageing. Therefore, further research in this area is area is warranted.

## 7 Limitations and Future Directions

In spite of a large number of drugs that are available to regulate the autophagic and UPR activity and some of them, indeed, have been licensed to be used in humans, like rapamycin, chloroquine and 4-PBA none of them were initially designed specifically to modulate autophagy and ER stress. Hence, the first obstacle that, so far, has prevented the current cellular homeostasis modulators from clinical treatment and deaccelerated their development, is the drug specificity. Almost all the chemical agents that are available currently to stimulate autophagy and UPR lack pharmacological specificity. That is, apart from their roles in the activation of these processes, they exert multiple non-specificity effects. For example, rapamycin and multiple rapalogues block the proliferation of T cells, exerting potent immunosuppressive effects (Baroja-Mazo et al., 2016). Questions also remain as to whether Tat-beclin-1 possess any non-specific actions resulting in unknown side-effects. Tat-Beclin-1 induces a dose-dependent and time-dependent effect. Although low doses of the peptide are non-cytotoxic, high dose have been shown to induce cell death in adult rat cardiomyocytes and human-induced pluripotent stem cells (Nah et al., 2020). One efficient way of increasing specificity is to focus on the components of the autophagic and UPR machinery that seem to have limited involvements in other processes, such as ATG4B (Akin et al., 2014) and ULK1 (Egan et al., 2015). Another mainstream strategy is to target selective pathways such as mitophagy (Sumpter et al., 2016). The current UPR regulating drugs do not directly target any upstream ER regulatory checkpoints. This is important as in a clinical setting, targeting these checkpoints has been identified as an optimal method to modulate ER stress and the UPR (Ren et al., 2021a).

Moreover, the architectural complexity of all tissues itself leads to specificity-related problems. Multiple types of cells within the tissue constitute complex homologous and heterologous interactions and the majority of current autophagy or UPR modulators do not specifically target a single cell type. Autophagy, mitophagy and the ER stress response occur in many tissues and cell types throughout the body. Therefore, pharmacological intervention may result in complete ablation or excessive activation of these pathways in multiple cell types and tissues meaning the desired therapeutic effect may not be achieved. Instead, targeting these processes in specific tissues or cell types is a viable approach to exploit the therapeutic value of the drugs (Galluzzi et al., 2017).

Currently, there are challenges in analysing the actual effect of the drugs at the cellular or even whole-body level. To address this complexity, the development of a suitable genetic model with cell-specific defects in specific components of the autophagic and UPR response is a viable strategy. The shortage of reliable and correct genetic models that can be used for the study of these pathways on disease pathogenesis and the development of novel agents is a main issue. In addition, the genetic models that have been used so far have several defects, like Atg4b^−/−^ mice, a genetic model with whole-body autophagy deficiency, still preserve some non-canonical autophagy response in several tissues including the liver, causing issues in the interpretation of disease progression before and after treatment in this kind of mice model (Niso‐Santano et al., 2015).

Alternatively, various tissue-specific deficiency models have been generated based on tissue specifically expressed Cre recombinase. However, these gene knockout models rely on the promoters of choice and they lead to autophagy and UPR defects at different stages of cell differentiation, thus causing considerable variability (Barinaga, 1994). Another issue related to this setting is the compensatory mechanism that may partially (if not totally) compensate for the absence of an individual protein, and this is the case for the autophagy and UPR machinery. To solve these issues, the conditional tissue-specific knockout model (rely on viral Cre delivery or pharmacological activation) has been introduced. But this model suffers from off-target problems that may accidentally lead to deficiencies in other tissues as well as the issue associated with the efficiency of Cre activation. Hence, taking the limitations of the current genetic models into consideration, further work is required to develop new models that bypass these persisting obstacles, facilitating the correct interpretation of discovery (Galluzzi et al., 2017).

Another related problem is the lack of standardised and reliable methods for discriminating the roles of different kinds of autophagy and UPR signalling, particularly *in vivo* and in patients (Klionsky et al., 2016). For example, the standard of current measurement of autophagy within the scientific community is still obscure. Therefore, considerable efforts are required for the generation of standardized methods that allow for monitoring of autophagy and UPR activity in distinct settings both *in vivo* and in patients, which will remove part of the current obstacles in the discovery and development of novel drugs.

The lack of translational research has significantly hindered the development of novel drugs involved in cellular homeostasis regulation. Although there have been advances in the understanding of the underlying signalling cascades within cells and novel therapeutic targets have been identified, few studies have investigated the drugs *in vivo* and the human setting. For example, although natural compounds hold great potential in modulating cellular homeostasis *in vitro*, a key drawback of their use is their poor bioavailability and solubility. Despite the cardioprotective benefits displayed by the various naturally occurring compounds, they possess a relatively low solubility leading to low oral absorption thus limiting therapeutic application. In addition, the long-term safety of many described drugs is also an area of concern. Therefore, further research to improve the clinical effect of these compounds is warranted (Xu et al., 2021).

## 8 Conclusion

Growing evidence from scientific studies supports the notion that there is an age-dependent decline in protein and cellular homeostasis which is mediated by impaired autophagy, mitophagy and UPR signalling. This imbalance promotes pathological cardiac ageing and increases the susceptibility of cardiac diseases, particularly heart failure. Decreased autophagy, mitophagy and UPR signalling result in the accumulation of damaged cellular elements and molecules such as organelle, misfolded proteins and lipids which threatens cell survival and cardiac function.

The mechanisms underlying autophagy, mitophagy and the UPR have been extensively investigated. Pharmacological intervention has shown to be able to maintain cellular equilibrium whereby limiting the damage associated with ageing and CVDs. Restoring efficient cellular homeostasis signalling is increasingly being recognized as an important therapeutic target. However, the central aim of promoting health and mitigating consequences of CVDs will require pharmacological interventions to be fine-tuned to ensure that the modulation of autophagy and UPR occurs in the correct cell type and tissue, at an appropriate time, and within a specific dose range to avoid excessive or inappropriate effects. To this end, efficiently and selectively targeting these crucial processes is vital to effectively utilize these drugs at their full potential. Multiple hurdles associated with the technical, experimental, pharmacological and translational aspects of drugs have significantly hampered their implementation into the clinical setting. These obstacles can be bypassed by the development of more selective drugs, suitable rodent models of autophagy and UPR deficiencies, standardisation of scientific methods and greater emphasis on translational research.
